# “Characteristics of children with multisystem inflammatory syndrome in children during different COVID-19 waves “Single centre Study”

**DOI:** 10.1186/s12887-025-06350-9

**Published:** 2025-11-25

**Authors:** Hanan M. Ibrahim, Alyaa kotby, Dalia H. El-Ghoneimy, Yasmin G. El Gendy, Iman Ragab, Hoda Tomom, Ahmed R. Rezk, Marwa W. Abdelhady, Abdelwahab Ahmed, Samuel Noshey, Samar A. Hassan, Sondos M. Magdy

**Affiliations:** 1 Pediatric Intensive Care Unit, Children’s Hospital, Ain Shams university, Cairo, Egypt; 2 Pediatric Cardiology Unit, Children’s Hospital, Ain Shams University, Cairo, Egypt; 3 Pediatric Allergy, Immunology and Rheumatology Unit, Children’s Hospital, Ain Shams University, Cairo, Egypt; 4 Pediatric Nutrition Unit, Children’s Hospital, Ain Shams University, Cairo, Egypt; 5 Pediatric Haematology and Oncology Unit, Children’s Hospital, Ain Shams University, Cairo, Egypt; 6 Pediatric Neurology Unit, Children’s Hospital, Ain Shams University, Cairo, Egypt

**Keywords:** Multisystem inflammatory syndrome in children (MIS-C), COVID-19, Pediatric patients, Clinical features, Cardiac involvement, Inflammatory markers, Treatment outcomes

## Abstract

**Background:**

Multisystem inflammatory syndrome in children (MIS-C) has been identified as a systemic inflammatory disorder complicating Coronavirus Disease 2019 (COVID-19) in children. Three different definition criteria have been proposed including clinical and laboratory features. MIS-C has variable presentation, severity and outcome. This study aimed to describe the clinic-laboratory and imaging features of MIS-C among Egyptian pediatric patients, as well as the treatment and outcome.

**Patients and methods:**

This was a retrospective observational study that included 142 MIS-C patients who were admitted at Children’s Hospital, Ain Shams University during the period from June 8, 2020, to January 26, 2022. Diagnosis of MIS-C followed CDC criteria. The medical records of the patients were reviewed for age, gender, initial presentation, interval time since COVID-19 infection or contact with COVID-19 patient, course of the disease, blood picture, acute phase reactants, kidney and liver function tests, imaging studies mainly echocardiogram and magnetic resonance imaging of the brain, therapeutic lines and outcome).

**Results:**

The median age varied from 4.5 years (4th wave) to 9 years (2nd wave). PCR positivity declined from 61% (1st wave) to 0% (4th wave), with lower IgG and higher IgM levels in the 1st wave (p < 0.001). Fever was universal (100%), Gastrointestinal symptoms were more common in the 1st wave (p = 0.005), whereas respiratory and neurological symptoms rose in later waves (p < 0.05). Lymphopenia and thrombocytopenia varied across waves (p < 0.01), with elevated inflammatory markers consistent throughout. Cardiac involvement, particularly coronary abnormalities, was highest in the 1st wave (p = 0.005). Treatment included IVIG and steroids, with increased use of pulse steroids and anti-IL1 therapy in later waves (p = 0.001). Hospital stays shortened from 10 days (2nd wave) to 3.5 days (4th wave), and mortality was highest in the 1st wave (9.4%). Conclusion: MIS-C cases in our center showed a consistent 2–7 weeks lag after COVID-19 peaks, with variable clinical and treatment trends. Improved outcomes over time, including shorter hospital stays and lower mortality.

## Introduction

Multisystem Inflammatory Syndrome in Children (MIS-C) is a rare but serious condition that complicates SARS-CoV-2 infection. First recognized in 2020, MIS-C typically develops 2–6 weeks after infection and is characterized by systemic inflammation with potential involvement of the cardiovascular, gastrointestinal, and other organ systems. Affected children often require hospitalization and, in severe cases, intensive care, underscoring the importance of early recognition and treatment [1, 2].

Case definitions from the World Health Organization (WHO) and Centers for Disease Control and Prevention (CDC) provide overlapping diagnostic criteria to support timely identification [3, 4]. More recently, the Council of State and Territorial Epidemiologists (CSTE) issued a revised case definition in 2023 to reduce complexity and misclassification, introducing standardized thresholds for inflammation and refining organ involvement criteria [5].

Although MIS-C incidence initially paralleled COVID-19 surges, reports have suggested a decline in cases despite the emergence of highly transmissible variants [6]. Data remain limited, however, on how clinical presentation, complications, and outcomes may have evolved with different waves of infection.

This study aimed to describe the clinical, laboratory, and echocardiographic characteristics, complications, and management of MIS-C cases across successive COVID-19 waves in Egypt.

## Materials and methods

This was a retrospective observational study, conducted at Children’s Hospital, Ain Shams University, Cairo, Egypt—a tertiary referral center with a dedicated isolation unit for children infected with COVID-19— at during the period between June 8, 2020, and January 26, 2022. MIS-C was diagnosed based on the US Centers for Disease Control and Prevention (CDC) definition [4]. Patients were excluded if any bacterial infection was diagnosed.

For each patient, the following data were collected (age, sex, clinical symptoms, laboratory results, echocardiographic data, possible complications, treatment, and outcome). COVID19 screening tests were done for all cases through SARS-CoV-2 reverse transcriptase (RT) PCR on nasopharyngeal and oropharyngeal swabs and serological testing for SARS-CoV-2 immunoglobulin G (IgG) and immunoglobulin M (IgM) (Epitope Diagnostics Inc, San Diego, California). Children included in the analysis were managed according to our adapted treatment protocol [8]. As per protocol the following treatments were given:

- IVIG dose (1–2 g/Kg) was divided into 2 days.
- corticosteroid dose was defined as methylprednisolone 1–2 mg/kg/day.
- Pulsed steroid therapy was defined as methylprednisolone dose of 10–30 mg/kg/day for 3 to 5 days.
- Anti-IL1 (Anakinra) dose: 1–2 mg/kg/day subcutaneous or intravenous infusion.
- Anticoagulant dose (Enoxaparin): 0.5 mg/kg/dose every 12 h (prophylactic dose) if D-dimer below 5 and if above 5 giving 1 mg/kg/dose every 12 h (therapeutic dose).

Patients were classified into low, moderate, or severe risk group as follows:

- Low-risk group: Children are looking mildly ill, presenting with fever, and symptoms of ≥ 2 organs involvement, with stable vital signs and no signs of cardiac dysfunction or hemodynamic instability.
- Moderate-risk group: Ill-appearing children, with fever, symptoms of 2 or more organs involvement (± cardiac involvement), and hemodynamically stable.
- Severe-risk group: Severely ill, toxic-appearing children, with evidence of shock or cardiac dysfunction and hemodynamic instability.

The classification of MIS-C severity into mild, moderate, and severe was based on our institutional algorithm previously published by Mahmoud et al. [7]. This practical approach has also been adopted in other institutional guidelines, including Children’s Minnesota [8] and the Maine AAP/Barbara Bush Children’s Hospital guideline [9], which stratify MIS-C severity into similar categories. Given that MIS-C severity is not uniformly defined in the literature, we chose this descriptive framework rather than applying sepsis-oriented scores such as pSOFA or PELOD-2, which are not MIS-C–specific [10].

The study was approved by the ethical committee of the Pediatric Department, Faculty of Medicine, Ain-Shams University. Written and informed consent was obtained from the parents. The data of the first wave was published in Mahmoud et al., 2021 [8], the remaining data has not been published before.

### Statistical analysis

Recorded data were analysed using the statistical package for social sciences, version 26.0 (SPSS Inc., Chicago, Illinois, USA). The quantitative data were presented as mean ± standard deviation and ranges when their distribution was parametric (normal) while non-normally distributed variables (non-parametric data) were presented as median with inter-quartile range (IQR). Also, qualitative variables were presented as number and percentages. Data were explored for normality using Kolmogorov-Smirnov and Shapiro-Wilk Test.

The following tests were done:

- A one-way analysis of variance (ANOVA) when comparing between more than two means & Post Hoc test: Tukey’s test was used for multiple comparisons between different variables.
- Kruskall Wallis test: for multiple-group comparisons in non-parametric data & Mann Whitney U test: for two-group comparisons in non-parametric data.
- The Comparison between groups with qualitative data was done by using Chi-square test and Fisher’s exact test instead of Chi-square test only when the expected count in any cell less than 5.

The confidence interval was set to 95% and the margin of error accepted was set to 5%. So, the p-value was considered significant if < 0.05.

##  Results

**Fig. 1:**
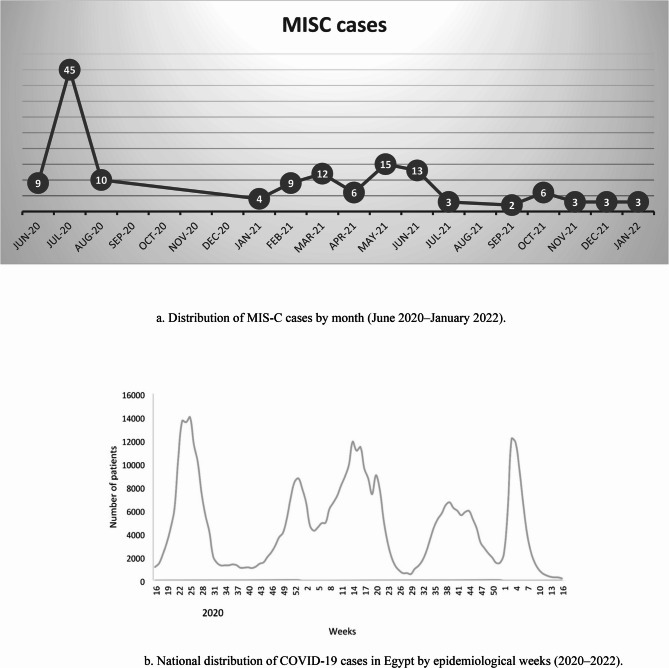
**a** Distribution of MIS-C cases by month (June 2020–January 2022). **b** National distribution of COVID-19 cases in Egypt by epidemiological weeks (2020–2022). Data source: Kandeel et al [12]

A total of 142 patients (85 males and 57 females) met the inclusion criteria and were included in the study. They were distributed across four waves, as shown in Fig. 1. Demographic data of the studied waves were detailed in Table 1, the lowest median age of the patients was in the fourth wave (4.5 years) with a p-value of 0.006.

**Table 1:** The demographic data, COVID-19 and clinical symptoms status of the studied groups

Demographic data	Wave 1 *(n = 64)*	Wave 2 *(n = 31)*	Wave 3 *(n = 31)*	Wave 4 *(n = 16)*	Test value	*p*-value
Age median (range)	7(0.08–14.08)	9(1–15)	7(1.5–14)	4.5(0.01–13.01)	5.432	0.006*
Sex						
Male	38 (59.4%)	12(38.7%)	14(45.2%)	5(31.3%)		
Female	26 (40.6%)	19(61.3%)	17(54.8%)	11(68.8%)	6.263	0.099
COVID PCR positive	39 (60.9%)	4(12.9%)	5(16.1%)	0(0.0%)	39.608	0.001**
COVID IGM	50 (78.1%)	9(29.0%)	6(19.4%)	2(12.5%)	46.042	0.001**
COVID IGG	17 (26.6%)	20(64.5%)	23(74.2%)	12(75.0%)	27.911	0.001**
Comorbidities	18 (28.1%)	6 (19.4%)	4 (12.9%)	2 (12.5%)	1.655	0.644
Fever before admission [days]	64 (100)	31 (100)	31 (100)	16 (100)		
Median (Range)	5 (3–10)	5(1–21)	5(1–16)	5(2–30)	4.367	0.224
Conjunctivitis	39 (60.9%)	24(77.4%)	30(96.8%)	16(100%)	20.795	0.001**
Rash/mucosal involvement (erythema or fissures, strawberry tongue)	58 (90.6%)	24(77.4%)	16(51.6%)	12(75.0%)	18.273	0.001**
Skin desquamation	35 (54.7%)	8(25.8%)	4(12.9%)	2(12.5%)	22.402	0.001**
Gastrointestinal symptoms	50 (78.1%)	15(48.4%)	15(48.4%)	8(50.0%)	12.914	0.005*
Respiratory symptoms	10 (15.6%)	15(48.4%)	9(29.0%)	6(37.5%)	11.940	0.008*
Hypotension	45 (70.3%)	17(54.8%)	23(74.2%)	8(50.0%)	4.953	0.175
Neurologic symptoms	4 (6.3%)	7(22.6%)	6(19.4%)	5(31.3%)	8.753	0.033*
Severity of illness						
* Mild-Moderate*	19 (29.7%)	10(33.3%)	9(29.0%)	9(56.3%)		
* Severe*	45 (70.3%)	21(67.7%)	22(71.0%)	7(43.8%)	4.451	0.2167
**p-value for multiple comparison between waves**						
	**P1 (W1, W2)**	**P2 (W1, W3)**	**P3 (W1, W4)**	**P4 (W2, W3)**	**P5 (W2, W4)**	**P6 (W3, W4)**
Age median (range)	0.234	0.678	0.093	0.456	0.014*	0.047*
COVID PCR positive	< 0.001**	< 0.001**	< 0.001**	0.707	0.144	0.136
COVID IgM	< 0.001**	< 0.001**	< 0.001**	0.373	0.197	0.551
COVID IgG	< 0.001**	< 0.001**	< 0.001**	0.393	0.457	0.957
Conjunctivitis	0.114	< 0.001**	0.003*	0.014*	0.024*	0.999
Rash/mucosal involvement (erythema or fissures, strawberry tongue)	0.069	< 0.001**	0.099	0.035*	0.853	0.106
Skin desquamation	0.007*	< 0.001**	0.003*	0.191	0.295	0.967
Gastrointestinal symptoms	0.003*	0.003*	0.029*	1.000	0.906	0.914
Respiratory symptoms	< 0.001**	0.123	0.057	0.111	0.474	0.548
Neurologic symptoms	0.023*	0.057	0.010*	0.752	0.512	0.328

Using: x2: Chi-square test for Number (%) or Fisher’s exact test, when appropriate

Kruskal–Wallis was performed for Median (Range) & Multiple comparison between groups through Mann-Whitney test

Different capital letters indicate significant difference at (p < 0.05) among the same rows

p-value > 0.05 is insignificant; *p-value < 0.05 is significant; **p-value < 0.001 is highly significant

^+^Gastrointestinal symptoms (vomiting, diarrhea, and abdominal pain), respiratory symptoms (tachypnea, cough, and oxygen requirement), neurological symptoms (headache, confusion, and convulsions)

P1: Wave 1 vs. Wave 2; P2: Wave 1 vs. Wave 3; P3: Wave 1 vs. Wave 4

P4: Wave 2 vs. Wave 3; P5: Wave 2 vs. Wave 4; P6: Wave 3 vs. Wave 4

COVID: Coronavirus Disease, PCR: Polymerase Chain Reaction, IgG Immunoglobulin G, IgM Immunoglobulin M.

Gastrointestinal symptoms were significantly more common in the 1 st wave (p-value = 0.005), we had three patients presented with severe abdominal pain with tenderness and rigidity on examination resembling acute abdomen one of them was operated as a case of acute appendicitis and postoperatively patient was deteriorating, developed cardiogenic shock and had elevated d-dimer, ferritin and C- reactive protein (CRP). His COVID serology was positive only for IgG. His echocardiography showed decreased ejection fraction (EF) 51%, mild tricuspid regurgitation (TR) and mitral regurgitation (MR) The patient was improved after receiving IVIG and pulse steroids therapy. The other 2 patients’ pelviabdominal computerized tomography (CT) examination revealed diffuse colitis that improved on medical treatment. Respiratory and neurological symptoms were least common in the 1 st wave (p-values = 0.008 and 0.033, respectively). Despite these differences, the severity of illness showed no significant variation across the four waves, as detailed in Table 1.

As regards laboratory findings (Table 2), lymphopenia present in 78/142 (55%) of the cohort and high inflammatory markers were evident as follows; high CRP 133/142 (93.6%), Hyperferritinemia 123/142 (86.6%) and high D-Dimer 122/142 (86%). The differences between the 4 waves are illustrated in Table 2.

**Table 2:** Comparison of selected laboratory results between the studied groups

Laboratory results	Wave 1 *(n = 64)*	Wave 2 *(n = 31)*	Wave 3 *(n = 31)*	Wave 4 *(n = 16)*	Test value	*p*-value
**High CRP**	64(100.0%)	25(80.6%)	28(90.3%)	16(100.0%)	14.844	0.002*
**Hyperferritinemia**	60(93.8%)	26(83.9%)	24(77.4%)	13(81.3%)	5.672	0.128
**Elevated D-dimer**	52(81.3%)	26(83.9%)	28(90.3%)	16(100.0%)	4.379	0.223
**High troponin**	40(62.5%)	8(25.8%)	9(29.0%)	1(6.3%)	25.039	0.001**
**Lymphopenia+**	45(70.3%)	13(41.9%)	15(48.4%)	5(31.3%)	12.391	0.006*
**Thrombocytopenia**	5(7.8%)	11(35.5%)	16(51.6%)	8(50.0%)	26.116	0.001**
**Thrombocytosis**	2(3.1%)	1(3.2%)	1(3.2%)	0(0.0%)	0.524	0.914
**Lymphocytic count**						
Median (IQR)	1.60 (0.93–2.10)	1.59(0.9–2.9)	1.4(1–2)	2.05(1.63–5.68)	1.881	0.160
Range	0.2–6.7	0.5–20	0.2–4.9	0.67–7.9		
**Platelet count**						
Median (IQR)	194.0 (91.5–325.5.5.5)	185(126–310)	145(98–244)	178(97.3–309)	1.067	0.349
Range	15–618	63–487	27–546	62–365		
**CRP**						
Median (IQR)	156.6 (47.0–300.0.0.0)	90(25–206)	168(106–270)	130(46.8–268.3.8.3)	1.295	0.280
Range	0.5–423	0–551	0–369	16–339		
**FERRITIN**						
Median (IQR)	513 (313–1222.5.5)	490(201.5–1080.8.5.8)	526(245–905)	199(90–1477.3.3)	0.113	0.893
Range	56.7–3049	0–2015	78–2688	0–4166		
**D DIMER**						
Median (IQR)	3.0 (1.99–4.15)	2(1.2–3.2)	2.8(0.825–5.6.825.6)	2.25(1.75–5.75)	0.631	0.535
Range	0.13–14.05	0.3–11.8	0.37–10.37	0.58–20.58		
**LDH**						
Mean ± SD	449.96 ± 128.63	303(215–389)	336(253.5–527.5.5.5)	311(261–347)	1.134	0.329
Range	175–969	165–565	201–1196	228–869		
**Troponin**						
Median (IQR)	0.08 (0.02–0.27)	0.017(0.002–0.14)	0.065(0.005–0.20.005.20)	0.013(0.001–0.028)	1.502	0.231
Range	0–2.1.1	0–1.03.03	0–4	0.001–0.77		
**p-value for multiple comparison between waves**						
	**P1** **(W1**,**W2)**	**P2** **(W1**,**W3)**	**P3** **(W1**,**W4)**	**P4** **(W2**,**W3)**	**P5** **(W2**,**W4)**	**P6** **(W3**,**W4)**
**High CRP**	< 0.001**	0.014*	1.000	0.273	0.044*	0.136
**High troponin**	< 0.001**	0.003*	< 0.001**	0.773	0.099	0.066
**Lymphopenia**	0.008*	0.038*	0.005*	0.607	0.475	0.258
**Thrombocytopenia**	< 0.001**	< 0.001**	< 0.001**	0.181	0.328	0.925

IQR: Interquartile range

Using: x2: Chi-square test for Number (%) or Fisher’s exact test, when appropriate

Using: One way Analysis of Variance test was performed for Mean ± SD & Multiple comparison between groups through Post Hoc test: Tukey’s test

Kruskal–Wallis was performed for Median (Range) & Multiple comparison between groups through Mann-Whitney test

Different capital letters indicate significant difference at (p < 0.05) among the same row

p-value >0.05 is insignificant; *p-value < 0.05 is significant; **p-value < 0.001 is highly significant

*PLT *Platelet, *CRP *C-reactive protein, *LDH *Lactate dehydrogenase

P1: Wave 1 vs. Wave 2; P2: Wave 1 vs. Wave 3; P3: Wave 1 vs. Wave 4

P4: Wave 2 vs. Wave 3; P5: Wave 2 vs. Wave 4; P6: Wave 3 vs. Wave 4

^+^ Lymphopenia was defined as low lymphocytic count that is normal to age group as defined by children’s reference ranges for FBC [28]

*CRP *C-reactive protein, *LDH *Lactate dehydrogenase.

Concerning cardiac involvement at presentation, elevated serum troponin levels were detected in 58 out of 142 patients (41%) (Table 2). The most common initial cardiac manifestation in our cohort was valvular involvement, predominantly affecting the tricuspid and mitral valves, though without statistically significant inter-group differences. Pulmonary regurgitation was identified in four patients during the first wave—including one case of severe regurgitation—and in two patients during the second wave. Aortic valve involvement was rare, with only two cases of mild regurgitation: one in the first wave and another in the fourth. Left ventricular systolic dysfunction was also frequent, with EF < 55% detected in up to 35% of cases, while LV dilatation occurred in 36.6% of patients, both without significant variation across waves (*p* = 0.623 and *p* = 0.668, respectively). Notably, coronary artery involvement was confined to wave 1, where 31.2% of patients had mild dilatation with < 2.5 Z- score, while it was absent in waves 2–4, yielding a highly significant overall difference (*p* = 0.001). Detailed data are presented in Table 3.

**Table 3:** Cardiac affection among studied population

Cardiac affection	Wave 1 *(n = 64)*	Wave 2 *(n = 31)*	Wave 3 *(n = 31)*	Wave 4 *(n = 16)*	Test value	*p*-value
**Ejection fraction%**						
**Below ≤ 55%**	20 (31.2%)	11 (35.4%)	14 (45%)	5 (31%)	1.08	0.623
Mean ± SD	56.44 ± 11.45	55.14 ± 13.57	54.54 ± 14.35	60.88 ± 11.06	1.269	0.288
Range	38–77	18–73	25–77	37–75		
**LV dilatation**						
Normal	42 (65.6%)	19(61.3%)	20(64.5%)	8(50.0%)	1.562	0.668
Abnormal	22 (34.4%)	12(38.7%)	10(32.3%)	8(50.0%)		
**Mitral regurgitation**						
Normal	31 (48.4%)	15(48.4%)	12(38.7%)	7(43.8%)	7.931	0.541
Mild	23 (35.9%)	13(41.9%)	15(48.4%)	8(50.0%)		
Moderate	8 (12.5%)	1(3.2%)	1(3.2%)	1(6.3%)		
Sever	2 (3.1%)	2(6.5%)	3(9.7%)	0(0.0%)		
**Tricuspid regurgitation**						
Normal	35 (54.7%)	10(32.3%)	10(32.3%)	5(31.3%)	21.758	0.009
Mild	12 (18.8%)	18(58.1%)	16(51.6%)	7(43.8%)		
Moderate	14 (21.9%)	3(9.7%)	3(9.7%)	4(25.0%)		
Severe	3 (4.7%)	0(0.0%)	2(6.5%)	0(0.0%)		
**Pericardial effusion**	7 (10.9%)	1(3.2%)	2(6.5%)	3(18.8%)	3.018	0.388
**Coronary artery affection**						
No	44 (68.8%)	31 (100%)	31 (100%)	16 (100.0%)	28.371	0.001**
Dilated	20 (31.2%)	0 (0.0%)	0 (0.0%)	0 (0.0%)		
**p-value for multiple comparison between waves**						
	**P1 (W1, W2)**	**P2 (W1, W3)**	**P3 (W1, W4)**	**P4 (W2, W3)**	**P5 (W2, W4)**	**P6 (W3, W4)**
**Coronary artery affection**	< 0.001**	< 0.001**	< 0.001**	1.000	1.000	1.000

Using: x2: Chi-square test for Number (%) or Fisher’s exact test, when appropriate

Using: One way Analysis of Variance test was performed for Mean ± SD & Multiple comparison between groups through Post Hoc test: Tukey’s test

Different capital letters indicate significant difference at (p < 0.05) among the same row

p-value > 0.05 is insignificant; *p-value < 0.05 is significant; **p-value < 0.001 is highly significant

*EF *Ejection fraction, *LV *Left ventricle, *MR *Mitral regurgitation, *TR *Tricuspid regurgitation, *CA *Coronary arteries

P1: Wave 1 vs. Wave 2; P2: Wave 1 vs. Wave 3; P3: Wave 1 vs. Wave 4

P4: Wave 2 vs. Wave 3; P5: Wave 2 vs. Wave 4; P6: Wave 3 vs. Wave 4

*Treatment plans across the four waves as shown in Table *4: In the first, second, and fourth waves, all patients received IVIG. In contrast, two patients in the third wave did not receive IVIG due to financial constraints. These patients had mild-to-moderate disease without cardiac involvement and experienced fever resolution after steroid treatment.

*Vasopressor and inotropic support:* Noradrenaline was the most used vasopressor for shocked patients across all four waves. Epinephrine was more frequently used as the initial inotropic support in the first and second waves but was not used in subsequent waves. Milrinone was preferred as the initial inotropic support in patients with arrhythmias or significant sinus tachycardia. Levosimendan was added in cases of severe cardiac dysfunction requiring higher inotropic support, which was more frequent in the third and fourth waves (*p* = 0.001).

*Anti-Inflammatory and anticoagulation therapies:* Anti-IL6 therapy was administered to two patients in the first wave, both of whom had significant underlying conditions and poor response to standard treatment.

In the second wave, one patient with severe disease and cardiogenic shock complicated by renal failure received anti-IL6 after no improvement with IVIG and steroids. As there was still no response, anti-IL1 was administered, followed by gradual cardiac recovery, discontinuation of inotropes and mechanical ventilation, and eventual cessation of dialysis.

**Table 4:** Comparison of management and outcomes between the studied groups

	Wave 1 *(n = 64)*	Wave 2 *(n = 31)*	Wave 3 *(n = 31)*	Wave 4 *(n = 16)*	Test value	*p*-value
IVIG	64(100.0%)	31(100.0%)	29(93.5%)	16(100.0%)	7.264	0.064
Steroids	60(93.8%)	31(100.0%)	31(100.0%)	14(87.5%)	6.091	0.107
High dose	21(32.8%)	27(87.1%)	27(87.1%)	10(62.5%)	38.674	0.001**
Low dose	39(60.9%)	4(12.9%)	4(12.9%)	4(25.0%)	32.503	0.001**
Milrinone/shocked	31/45(68.9%)	18/19(94.7%)	1723/(73.9%)	7/8(87.5%)	1.298	0.729
Levosimendan/shocked	0/45(0.0%)	1/19(5.3%)	7/23(30.4%)	4/8(50.0%)	20.666	0.001**
Epinephrine/shocked	12/45(26.7%)	4/19(21.1%)	0/23(0.0%)	0/8(0.0%)	9.635	0.021*
Noradrenaline/shocked	43/45(95.6%)	1/19(5.3%)	21/23(91.3%)	7/8(87.5%)	3.629	0.304
Biological therapy						
Anti IL1	0(0.0%)	9(29.0%)	8(25.8%)	4(25.0%)	20.408	0.001**
Anti IL 6	2(3.2%)	1(3.2%)	0(0.0%)	0(0.0%)	1.52	0.678
Anticoagulation Therapy	52(81.3%)	28(90.3%)	23(74.2%)	10(62.5%)	5.737	0.125
Aspirin	35(54.7%)	1(3.2%)	7(22.6%)	5(31.3%)	27.219	0.001**
LOS (days), median (range)	9 (4–46)	10(5–20)	7(2–22)	3.5(2–20)	3.124	0.050*
Intubation, n [%]	13(20.3%)	1(3.2%)	4(12.9%)	1(6.3%)	6.120	0.106
Neurological complications	0(0.0%)	5(16.1%)	2(6.5%)	1(6.3%)	10.294	0.016*
Outcome						
Discharged	58(90.6%)	30(96.8%)	30(96.8%)	15(93.8%)		
Died	6(9.4%)	0(0%)	1(3.2%)	1(6.3%)	3.89	0.274
**p-value for multiple comparison between waves**						
	**P1 , W2)**	**P2 (W1, W3)**	**P3 (W1, W4)**	**P4 (W2, W3)**	**P5 (W2, W4)**	**P6 (W3, W4)**
**High dose**	< 0.001**	< 0.001**	0.025*	1.000	0.041*	0.041*
**Low dose**	< 0.001**	< 0.001**	0.009*	1.000	0.272	0.272
**Levosimendan/shocked**	0.143	< 0.001**	< 0.001**	0.035*	0.007	0.297
**Epinephrine/shocked**	0.634	0.004*	0.077	0.035	0.133	1.000
**Biological therapy: Anti IL1**	< 0.001**	< 0.001**	< 0.001**	0.774	0.808	0.958
**Aspirin**	< 0.001**	0.004*	0.098	0.035*	0.016*	0.522
**LOS (days)**	0.234	0.004*	< 0.001**	< 0.001**	< 0.001**	0.047*
**Neurological complications**	0.002*	0.077	0.206	0.235	0.363	0.972

Using: x2: Chi-square test for Number (%) or Fisher’s exact test, when appropriate

Kruskal–Wallis was performed for Median (Range) & Multiple comparison between groups through Mann-Whitney test

Different capital letters indicate significant difference at (*p* < 0.05) among the same row

*p*-value > 0.05 is insignificant; *p-value < 0.05 is significant; **p-value < 0.001 is highly significant

IVIG: intravenous immunoglobulin, LOS: length of stay

### Morbidities and mortality

Neurological complications were most commonly observed during the second wave, followed by the third and fourth waves. In the second wave, five patients developed neurological symptoms: one patient presented with squint, another with blindness—MRI findings in the latter were consistent with posterior reversible encephalopathy syndrome (PRES). Another patient experienced severe headache due to increased intracranial pressure, while two patients developed aphasia—one with a normal MRI and the other showing a midbrain infarction. In the third and fourth waves, neurological complications occurred in three patients, all of whom presented with severe headaches and had MRI findings suggestive of central nervous system (CNS) vasculitis. Notably, all these neurological manifestations appeared after the resolution of initial shock and an early response to treatment.

Mortality occurred in 8/142 patients (5.6%), 7 patients of them had associated comorbidities 5 occurred in children with significant comorbidities, including congenital heart disease (1 patient), polyarteritis nodosa (1 patient), and hematologic malignancy (3 patients), 2 of whom were below 6 months of age. The main causes of mortality were refractory shock and multi-organ failure despite maximal supportive therapy. One patient presented very late with sever cardiogenic shock, pulmonary edema and arrythmia she did not respond to treatment with IVIG, pulse steroid therapy and continuous IV infusion of anakinra and after 2 days unfortunately she died.

## Discussion

Egypt’s first wave of the COVID-19 pandemic occurred between April and September 2020 and was predominantly associated with B lineages, particularly B.1, consistent with global trends at that time. Subsequent waves saw the emergence of C.36 and C.36.3 lineages, followed by dominance of the Delta variant (B.1.617.2) during the fourth wave and Omicron (B.1.1.529) during the fifth wave [11]. In our cohort, patients were distributed across four waves, aligning with the epidemiological pattern reported by Kandeel et al. [12], who documented five distinct SARS-CoV-2 waves in Egypt between March 2020 and April 2022.

A consistent lag of 2–7 weeks was observed between peaks of COVID-19 infections and the onset of MIS-C cases, highlighting the temporal relationship between acute infection and the subsequent inflammatory syndrome. This is in keeping with WHO [3] and CDC [4] definitions of MIS-C, which recognize the post-infectious timing of onset.

We observed a progressive decline in MIS-C cases over time, from 64 in the first wave to 31 in the second and third waves combined, and only 16 in the fourth wave. This reduction occurred despite the increasing transmissibility of later SARS-CoV-2 variants. Similar trends were reported by Ptak et al. [6], who found fewer admissions during Delta/Omicron periods than during the original/Alpha wave, and by Levy et al. [13], who attributed lower incidence during Omicron to variant characteristics, prior infections, and vaccination. Recent immunological studies [14] suggest that Omicron may induce less systemic immune activation compared with earlier variants, possibly due to altered spike protein interactions and reduced superantigen-like activity, leading to lower MIS-C risk. Epidemiologically, widespread immunity from previous infection and vaccination likely further attenuated the hyperinflammatory response characteristic of MIS-C.

Together, these findings suggest that population-level immunity, through both natural infection and vaccination, likely contributed to declining MIS-C incidence globally.

Age distribution varied significantly, with the highest median age (9 years) during the second wave and the lowest (4.5 years) in the fourth wave, while no consistent gender predominance was identified. This aligns with prior studies reporting median ages between 8 and 11 years [15] and the large systematic review by Abbas et al. [16], which included 20,881 cases and reported a median age of 7.8 years with a male predominance.

Virological and serological profiles also differed by wave. The first wave had the highest COVID-19 IgM positivity and PCR detection, with the lowest IgG positivity, reflecting naïve immune responses early in the pandemic. In later waves, PCR positivity fell sharply (13% and 16% in the second and third waves, respectively, and 0% in the fourth). This pattern is consistent with prior reports [17, 18] and may be explained by both higher background immunity and limitations of available diagnostic assays in detecting emerging variants.

Fever remained a universal feature across all waves, consistent with MIS-C diagnostic criteria [4], with median duration of five days, aligning with prior studies (4–6 days) [15]. However, respiratory and neurological symptoms became more prominent in later waves, potentially reflecting variant-specific tropisms or shifts in immune response. Ptak et al. [6] similarly noted more respiratory involvement during Omicron, while neurological manifestations are increasingly recognized as part of MIS-C pathophysiology [19, 20]. Sa et al. [19] reported higher inflammatory markers in MIS-C patients with neurological involvement, supporting an immune-mediated mechanism.

Laboratory abnormalities also varied. Lymphopenia was most common in the first wave, possibly reflecting a stronger hyperinflammatory response before widespread immunity was established [15, 21]. Thrombocytopenia was also observed, consistent with hyperinflammation and endothelial activation. Unlike Kawasaki disease (KD), which typically shows thrombocytosis, severe KD can present with thrombocytopenia and higher risk of coronary aneurysm [22].

Cardiac involvement was frequent, with elevated troponin levels particularly common in the first wave, suggesting more severe myocardial injury early in the pandemic. Elevated troponin is now formally recognized as a diagnostic criterion for MIS-C cardiac involvement under the 2023 CDC case definition [23]. Our most frequent echocardiographic abnormalities were valve regurgitation (66%), followed by left ventricular dilatation (36.6%), low ejection fraction (35%), and coronary involvement (19.7%). This contrasts with KD, where coronary artery dilation predominates, although ventricular dysfunction can occur during acute illness [15, 24].

Cardiac involvement in MIS-C was prominent in our cohort, with elevated troponin levels detected particularly during the first wave—suggesting that myocardial injury tended to be more pronounced in early pandemic phases. This finding aligns with emerging diagnostic frameworks, such as the 2023 CDC case definition [23], which now formally includes troponin elevation as evidence of cardiac involvement in MIS-C.

Our most frequent echocardiographic abnormalities were valve regurgitation (66%), followed by left ventricular dilatation (36.6%), reduced ejection fraction (35%), and coronary artery involvement (19.7%). In comparison, a recent systematic review [24] of 1,392 MIS-C patients across 33 studies found left ventricular systolic dysfunction in approximately 34.9%, valvular regurgitation in 29.1%, pericardial involvement in 22.6%, and coronary abnormalities in 18.0%. These values are broadly consistent with our findings regarding ventricular dysfunction and coronary involvement, though our rate of valvular regurgitation is substantially higher. This difference may reflect population-specific factors or variations in echocardiographic protocols and definitions.

By contrast, in Kawasaki disease (KD), coronary artery dilation tends to be the hallmark feature, although ventricular dysfunction and valvular regurgitation can occur during acute stages [15, 25]. This reinforces the concept that while MIS-C and KD share features, MIS-C may present with a wider range of myocardial and valvular pathology.

Treatment patterns evolved across waves. According to hospital protocol, all patients with severe disease received IVIG and steroids. The nearly universal use of IVIG mirrors international guidelines [4]. Two patients in the third wave who did not receive IVIG due to socioeconomic barriers recovered with steroids alone, highlighting that mild disease may sometimes respond to steroids only [15]. The increasing use of pulse corticosteroid therapy in later waves reflects a transition to more aggressive immunomodulation as clinical experience accumulated [26].

Hemodynamic support also shifted over time. Whereas epinephrine was commonly used initially, later practice favoured milrinone and Levosimendan, reflecting recognition that MIS-C-related myocardial dysfunction may predispose patients to epinephrine-induced tachyarrhythmias. Indeed, several patients developed arrhythmias after epinephrine initiation.

Biologic therapies were reserved for refractory cases. Following the American College of Rheumatology 2020 recommendations [27], anakinra was used in severe cases resistant to IVIG and corticosteroids (21 patients, 14.7%), reflecting broader global practice.

Clinical outcomes improved over time, with shorter hospital stays and lower mortality in later waves. This likely reflects earlier recognition, refined management strategies, and increased immunity. The higher mortality in the first wave, particularly among children with comorbidities, underscores the challenges of managing a novel syndrome at the onset of the pandemic [15].

### Novelty of our findings

Our cohort demonstrates declining MIS-C incidence despite more transmissible variants, a predominance of valvular regurgitation rather than coronary changes, and shifting clinical phenotypes across waves. Together, these findings add to the global literature by highlighting the heterogeneity of MIS-C cardiac involvement and underscoring the importance of regional experience in shaping management strategies.

### Limitations

This study has several limitations that should be considered when interpreting the findings. First, it was conducted at a single center, which may limit the generalizability of the results. Second, the sample size was relatively small, reducing the power to detect subtle differences between waves. Third, the study did not adjust for potential confounders, such as changes in clinical protocols, immunomodulatory strategies, diagnostic testing capacity, and background immunity, all of which may have influenced the observed differences.

Another limitation is that we did not perform statistical correlation between specific cardiac abnormalities (e.g., EF reduction, valvular insufficiency, coronary changes) and outcomes such as length of PICU stay or mortality, which could be an area for future research.

Finally, long-term outcomes were not assessed, which could have provided additional insights into the evolving burden of MIS-C across successive waves.

## Conclusion

Cases of MIS-C in our center demonstrated a declining trend across successive COVID-19 waves, despite the emergence of more transmissible variants. This decline, accompanied by shifts in age distribution, clinical manifestations, and laboratory findings, likely reflects evolving population immunity, earlier recognition, and improved treatment protocols.

Future Directions:

Further large-scale, multicentre studies with standardized protocols and adjustment for potential confounders are needed to confirm these findings and to better define the long-term outcomes of MIS-C across different populations and settings.

## Data Availability

The datasets used and/or analyzed during the current study are available from the corresponding author on reasonable request.

## Abbreviations

ANOVA: Analysis of Variance
CDC: Centers for Disease Control and Prevention
CNS: Central Nervous System
COVID-19: Coronavirus Disease 2019
CRP: C-Reactive Protein
CSTE: Council of State and Territorial Epidemiologists
CT: Computed Tomography
EF: Ejection Fraction
IgG: Immunoglobulin G
IgM: Immunoglobulin M
IL1: Interleukin-1
IL6: Interleukin-6
IQR: Interquartile Range
IVIG: Intravenous Immunoglobulin
KD: Kawasaki Disease
MIS-C: Multisystem Inflammatory Syndrome in Children
MRI: Magnetic Resonance Imaging
PCR: Polymerase Chain Reaction
RT: Reverse Transcription
SARS-CoV-2: Severe Acute Respiratory Syndrome Coronavirus 2
VOC: Vaso-Occlusive Crisis
WHO: World Health Organization
